# CD19-negative non-IgM type lymphoplasmacytic lymphoma: a case report and literature review

**DOI:** 10.3389/fonc.2026.1809955

**Published:** 2026-07-13

**Authors:** Feng Lu, Fugang Li, Hongying Zhan, Yuanfeng Huang, Rong Huang, Xuchuan Jiang, Chengjie Ji, Gang Wang, Tao Chen, Xia Fu, Tao Wei, Weijie Jiang

**Affiliations:** The People’s Hospital of Jianyang City, Jianyang, Sichuan, China

**Keywords:** flow cytometry, immunophenotype, lymphoplasmacytic lymphoma, monoclonal immunoglobulin, non-IgM type lymphoplasmacytic lymphoma

## Abstract

Lymphoplasmacytic lymphoma (LPL) that does not fulfill the diagnostic criteria for Waldenström’s macroglobulinemia (WM), termed non-IgM type LPL, represents a rare entity that poses significant diagnostic challenges. Here, we report a rare case of CD19-negative non-IgM type LPL. A 69-year-old man was incidentally found to have elevated M-protein and globulin levels during a routine health examination. Subsequent evaluation revealed elevated serum IgG, an IgG-λ monoclonal protein, and markedly elevated urinary λ free light chains. Bone marrow biopsy demonstrated normocellular marrow with approximately 40% cellularity. Immunohistochemistry revealed that CD20^+^ B lymphocytes predominated over CD3^+^ T lymphocytes, exhibiting an interstitial infiltration pattern. PAX5 staining highlighted B lymphocytes, comprising approximately 30% of total nucleated cells. CD138 staining revealed plasma cells in a scattered and clustered distribution, accounting for 3−5% of nucleated cells. Flow cytometry identified a monoclonal B lymphocyte population that was CD19-negative and CD20-positive, with restricted λ light chain expression, coexisting with a monoclonal plasma cell population showing restricted cytoplasmic λ light chain expression. Although the bone marrow biopsy lacked characteristic lymphoid aggregates, molecular testing confirmed the presence of the *MYD88* L265P mutation and the absence of the *CXCR4* mutation. The integration of morphological, immunophenotypic, and molecular findings with clinical features established the diagnosis of CD19-negative non-IgM type LPL. This case underscores the critical role of a comprehensive, multi-platform diagnostic approach and effective clinician-laboratory communication in accurately diagnosing hematologic malignancies with atypical immunophenotypes.

## Introduction

Lymphoplasmacytic lymphoma (LPL) is a rare, indolent mature B-cell lymphoma, accounting for less than 2% of all non-Hodgkin lymphomas. Pathologically, LPL is characterized by bone marrow infiltration (often involving lymph nodes and the spleen) by a clonal proliferation of small B lymphocytes, plasmacytoid lymphocytes, and plasma cells. The diagnosis is established only after excluding other small B-cell lymphomas with plasmacytic differentiation (1–3). Clinically, most LPL cases are characterized by IgM paraprotein secretion and are classified as Waldenström’s macroglobulinemia (WM). However, approximately 5% of LPL patients present with a non-WM phenotype, secreting IgG, IgA, or no monoclonal immunoglobulin (2). Although plasma cells in most LPL cases typically express CD19 alongside clonal B cells, literature confirms that a subset of LPL cases harbors CD19-negative clonal plasma cells (4). This atypical immunophenotype poses significant diagnostic challenges, as initial flow cytometry panels designed primarily to screen for plasma cell disorders may inadvertently miss these CD19-negative clonal populations, leading to potential misdiagnosis. Herein, we report a rare case of CD19-negative non-IgM type LPL, characterized by the concurrent loss of CD19 expression on both clonal B cells and plasma cells. This case reaffirms that no single test is definitive; rather, an accurate diagnosis relies on the comprehensive integration of morphological assessment, multiparameter flow cytometry, and molecular testing.

## Case presentation

A 69-year-old man presented to our hospital for a routine health examination on May 12, 2025, during which elevated serum globulin and M-protein levels were incidentally detected. Immunofixation electrophoresis identified an IgG-λ monoclonal protein. Consequently, the patient was admitted to the Department of Hematology on May 23, 2025, for further evaluation. On admission, physical examination was unremarkable; specifically, no superficial lymphadenopathy or hepatosplenomegaly was palpable. Ultrasonography confirmed the absence of significant lymphadenopathy in the cervical, axillary, inguinal, or retroperitoneal regions.

Serum protein electrophoresis revealed an M-protein spike constituting 30.4% of total protein (reference range: 8.4%-13.1%), corresponding to an estimated concentration of 2.69 g/dL. Routine biochemistry showed elevated total protein (88.5 g/L; reference range: 65–85 g/L) and globulin (45.1 g/L; reference range: 20–40 g/2L), with a normal albumin level of 43.4 g/L. Complete blood counts were unremarkable: white blood cells (WBC) 4.62 × 10^9^/L, red blood cells (RBC) 4.78× 10^12^/L, hemoglobin (HGB) 159 g/L, and platelets (PLT) 169 × 10^9^/L. Serum immunoglobulin quantification showed marked elevation of IgG (28.0 g/L; reference range: 8.6–17.4 g/L) and decreased IgA (0.52 g/L; reference range: 1–4.2 g/L), with normal IgM levels (0.33 g/L; reference range: 0.3–2.2 g/L). Urinary light chain analysis demonstrated marked elevation of the λ chains (277.89 mg/L; reference range: 0–15 mg/L), contrasting with a normal κ chain levels (6.75 mg/L; reference range: 0–15.25mg/L).

**Figure 1:**
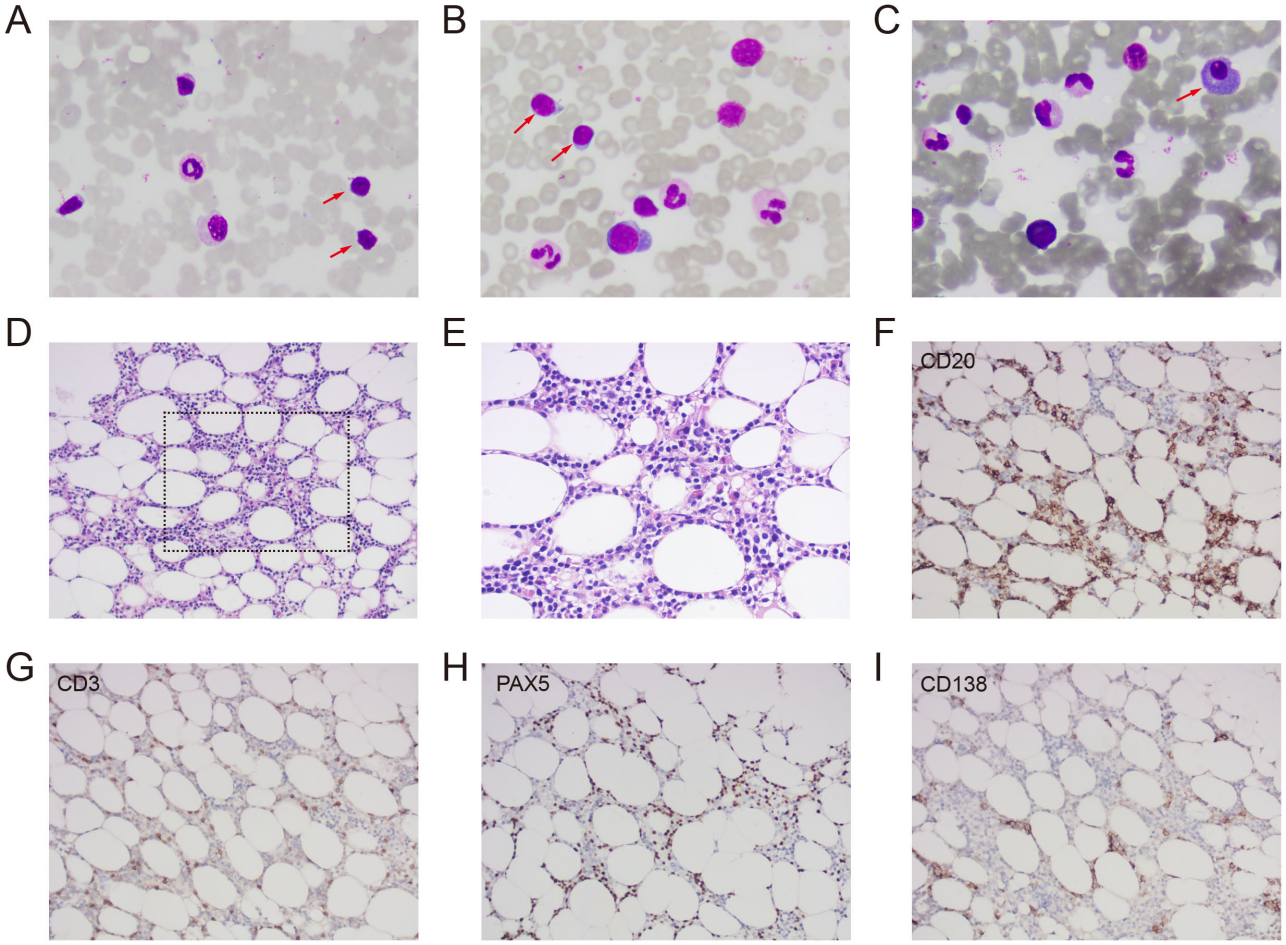
Morphological and immunohistochemical findings of bone marrow involvement. **(A-C)** Bone marrow aspirate smears (Wright-Giemsa stain, ×1000). **(A)** Small lymphocytes (arrow): small, round to ovoid cells with scant pale blue cytoplasm. **(B)** Plasmacytoid lymphocytes (arrow): slightly larger than small lymphocytes, featuring more abundant cytoplasm and eccentric nuclei. **(C)** Plasma cells (arrow). **(D, E)** Bone marrow core biopsy (H&E stain), **(D)** ×200 magnification; **(E)** ×400 magnification, showing no discrete lymphoid aggregates. **(F-I)** Immunohistochemical staining of the bone marrow core biopsy (×200 magnification). **(F)** CD20 highlights B lymphocytes with an interstitial infiltration pattern. **(G)** CD3 highlights scattered T lymphocytes. **(H)** PAX5 shows nuclear positivity in B lymphocytes, comprising approximately 30% of nucleated cells. **(I)** CD138 shows plasma cells in a scattered and clustered distribution.

Bone marrow aspirate smears demonstrated normocellularity with a myeloid-to-erythroid ratio of 3.29:1. The morphology of granulocytic and erythroid lineages was unremarkable. The lymphocyte proportion was elevated at 34.5%, predominantly consisting of small lymphocytes. Morphologically, these lymphocytes were small and round to oval with scant pale blue cytoplasm and nuclei containing coarsely clumped chromatin. A subset of slightly larger cells displayed plasmacytoid features, characterized by more abundant cytoplasm and eccentric nuclei with clumped chromatin. Plasma cells accounted for 1% of nucleated cells (**Figures 1A–C**). Routine hematoxylin and eosin (H&E) staining of the bone marrow biopsy revealed normocellular marrow with approximately 40% cellularity, normal trilineage hematopoiesis, scattered lymphocytes and plasma cells, and no discrete lymphoid aggregates (**Figures 1D, E**).

No significant reticulin fibrosis was observed (MF-0 grade). Immunohistochemical stains were conducted to further characterize the infiltrate, which appeared subtle on routine H&E morphology. These stains revealed that CD20^+^ B lymphocytes predominated over CD3^+^ T lymphocytes, exhibiting an interstitial infiltration pattern. PAX5 staining highlighted B lymphocytes, comprising approximately 30% of total nucleated cells. CD138 staining identified plasma cells in both scattered and clustered distributions, accounting for approximately 3%–5% of nucleated cells (**Figures 1F–I**); notably, these plasma cells were negative for CD117.

**Figure 2:**
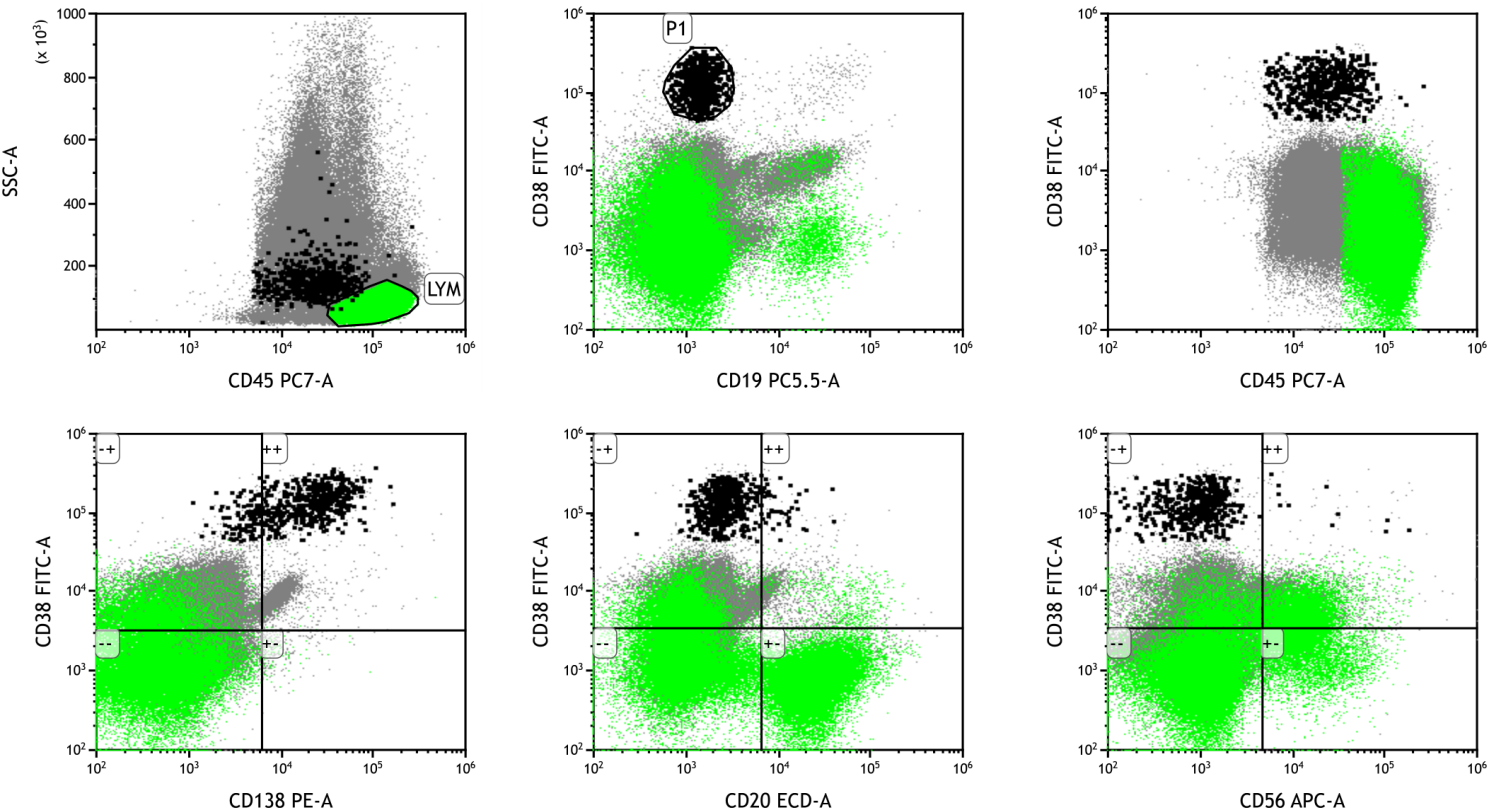
Aberrant surface immunophenotype of plasma cells detected by flow cytometry. The black cell population within gate P1 represents aberrant plasma cells expressing CD38 and CD138, but negative for CD19, CD20, and CD56 (cytoplasmic κ and λ staining results are shown in **Figure 3A**).

**Figure 3:**
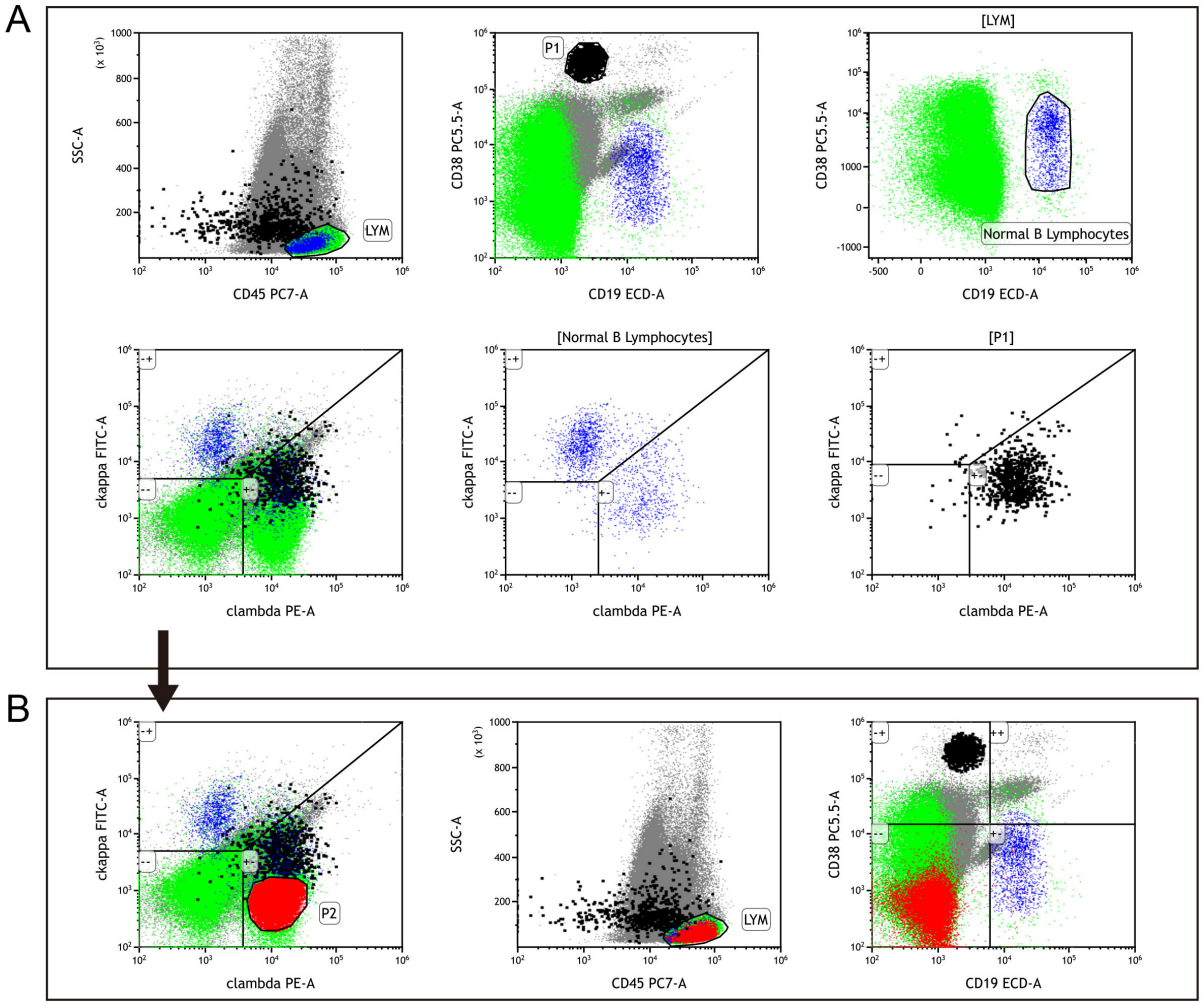
Flow cytometric immunophenotyping – identification of monoclonal plasma cells and discovery of the clonal B-cell population. **(A)** Monoclonal plasma cells (black, gate P1) express CD38 with cytoplasmic λ light chain restriction, while lacking CD19 and cytoplasmic κ light chain. **(B)** Reverse gating from cytoplasmic λ-positive cells within the lymphocyte gate (CD38-negative) identified a distinct population (red, gate P2) that lacked CD19 and CD38 but expressed λ light chain. This finding prompted additional B-cell lymphoma panel testing (see **Figure 4**).

**Figure 4:**
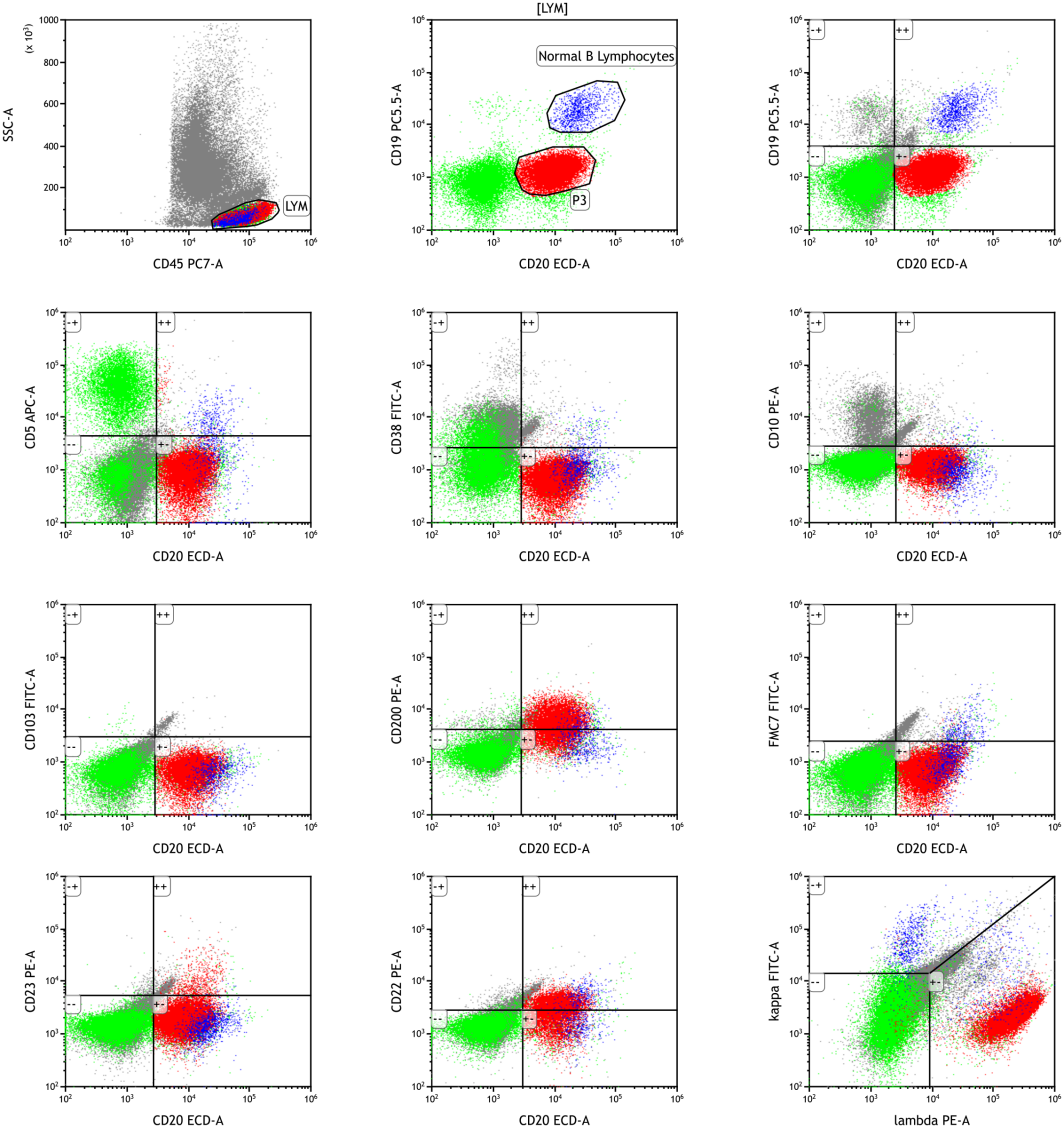
Flow cytometric immunophenotyping of monoclonal B lymphocytes. The red cell population within gate P3 represents the monoclonal B lymphocyte subset. These cells express CD20 and λ light chain, partially express CD22 and CD200, and are negative for CD5, CD10, CD19, CD23, CD38, CD103, FMC7, and κ light chain.

Initial flow cytometric screening, primarily aimed at evaluating for multiple myeloma, detected a minor population (approximately 0.5%) of monoclonal plasma cells. These cells expressed CD38 and CD138 with restricted cytoplasmic λ light chain expression but lacked CD19, CD20, CD56, and cytoplasmic κ light chain (**Figures 2**, **3A**). However, critical re-evaluation of the flow data revealed a distinct population within the lymphocyte gate that expressed cytoplasmic λ light chain but lacked CD19 and CD38 (**Figure 3B**). Subsequent expanded workup for mature B-cell non-Hodgkin lymphoma identified a population of monoclonal B lymphocytes, comprising approximately 12% of nucleated cells. These cells were CD19-negative, CD20-positive, and partially expressed CD22 and CD200. They demonstrated restricted λ light chain expression and were negative for CD5, CD10, CD23, CD38, CD103, FMC7, and κ light chain (**Figure 4**). Residual polytypic CD19^+^ B cells accounted for approximately 1.1% of nucleated cells, yielding an abnormal-to-normal B−cell ratio of roughly 11:1.

Molecular studies were positive for the *MYD88* L265P mutation by allele-specific PCR, whereas no *CXCR4* mutation was identified by Sanger sequencing.

Based on the integrated morphological, immunophenotypic, molecular, and clinical findings, a diagnosis of non-IgM type LPL was established. Given that the patient’s presentation was limited to elevated serum IgG levels without symptoms of fatigue, B symptoms, hyperviscosity, neuropathy, or significant lymphadenopathy, a strategy of active surveillance without immediate intervention was recommended.

Follow−up information was available as of April 18, 2026, approximately 11 months post-diagnosis. Biochemical assessment showed a serum total protein of 88.8 g/L (reference range: 65–85 g/L) and a globulin level of 50.2 g/L (reference range: 20–40 g/L). Complete blood counts, serum immunofixation, and imaging studies were not performed at this time point. The patient remained clinically stable, with no evidence of B symptoms, lymphadenopathy, or hepatosplenomegaly. In the absence of clinical or laboratory signs of disease progression, the strategy of active surveillance was continued.

## Discussion

The diagnostic trajectory in this case commenced with the incidental detection of an elevated serum M-protein, identified by immunofixation electrophoresis as monoclonal IgG-λ. Subsequent evaluation revealed elevated serum IgG, decreased IgA, and a marked increase in urinary λ light chains. The initial flow cytometric screening, primarily aimed at evaluating for multiple myeloma, detected a minor population of monoclonal plasma cells exhibiting restricted cytoplasmic λ light chain expression. However, a critical re-evaluation of the flow data uncovered a distinct population within the lymphocyte gate that expressed cytoplasmic λ light chain but lacked CD19 and CD38. This finding prompted an expanded workup for mature B-cell non-Hodgkin lymphoma, which identified a dominant population of monoclonal B lymphocytes. These cells were CD19-negative and CD20-positive, partially expressed CD22 and CD200, and demonstrated restricted λ light chain expression, while lacking other B-cell markers including κ light chain. The co-existence of phenotypically aberrant, clonally concordant B lymphocytes and plasma cells posed a significant diagnostic dilemma. Furthermore, bone marrow cytology revealed lymphocytosis with infiltration by small lymphocytes, plasmacytoid lymphocytes, and plasma cells. Nevertheless, the pathological manifestations in this patient were atypical, characterized by the absence of obvious lymphoid aggregates, increased mast cells, or hemosiderin deposition. Ultimately, the discordance between the nonspecific morphological presentation and the rare CD19-negative immunophenotype rendered the diagnosis particularly challenging.

Consequently, the differential diagnosis focused on four distinct entities. First, plasma cell neoplasm (PCN) was the primary consideration given the presence of an IgG-λ paraprotein and clonal plasma cells. However, several features argued against this diagnosis: the absence of lytic bone lesions, normal calcium levels and renal function, CD56 negativity on plasma cells, negative CD117 immunohistochemistry, and partial retention of CD45 expression. Most critically, the presence of a prominent clonal B-cell population combined with the *MYD88* L265P mutation rendered a diagnosis of classical PCN highly unlikely, as *MYD88* mutations are virtually absent in such cases (5). Second, non-IgM monoclonal gammopathy of undetermined significance (MGUS) was included in the differential diagnosis given the patient’s asymptomatic status, an M-protein concentration of approximately 2.6 g/dL, bone marrow clonal plasma cells <10%, and the absence of end-organ damage. Typically, non-IgM MGUS represents a precursor lesion of multiple myeloma arising from the plasma cell lineage (6, 7). However, data from the iSTOPMM study indicate that among 37 cases of non−IgM MGUS, 25 (68%) exhibited clonal plasma cells only, 9 (24%) showed concurrent clonal plasma cells and clonal B−lymphocytes, and 1 (3%) presented with clonal B−lymphocytes only (8). These findings suggest that our case, characterized by both clonal populations in the bone marrow, cannot be definitively distinguished from non-IgM MGUS based on flow cytometry alone. Nevertheless, in our case, the bone marrow infiltration was predominantly B-cell in nature, and flow cytometry confirmed that the clonal B cells and plasma cells shared identical λ light chain restriction. While these features favor a diagnosis of non-IgM LPL, we acknowledge that this case may represent a “gray zone” between non−IgM MGUS and non−IgM LPL, necessitating continued clinical follow-up. Third, although marginal zone lymphoma (MZL) can exhibit plasmacytic differentiation and occasionally harbor *MYD88* mutations, this diagnosis was considered unlikely in our patient due to the absence of splenomegaly and the lack of paratrabecular or sinusoidal infiltration on bone marrow biopsy. Additionally, the neoplastic B cells appeared as monotonous small lymphocytes, lacking the cellular heterogeneity typically observed in MZL. Combined with the confirmatory *MYD88 L265P* mutation, these findings rendered MZL less likely (9). Fourth, chronic lymphocytic leukemia/small lymphocytic lymphoma (CLL/SLL) with plasmacytic differentiation was excluded based on the absence of CD5 and CD23 expression on B cells, the lack of proliferation centers on biopsy, and the absence of peripheral blood lymphocytosis. Finally, referring to expert consensus on the application of flow cytometry in mature B-cell lymphoma diagnosis (10, 11), and the molecular profile characterized by *MYD88* positivity and *CXCR4* negativity supported the diagnosis of LPL.

Notably, the complete absence of CD19 on both clonal B−cell and plasma cell populations is a rare finding in LPL. Most published series report CD19 expression on B cells in 100% of cases and on plasma cells in 96% of non-IgM LPL cases (5). Nevertheless, rare CD19-negative cases do exist, as illustrated by our patient and two previously reported cases of IgG-type LPL with CD19-negative plasma cells (4). This atypical immunophenotype can lead to diagnostic delays, given that CD19 is commonly employed as a primary gating marker for B-cell populations. In our case, the initial flow cytometry panel was oriented toward multiple myeloma, resulting in the initial oversight of the CD19-negative B-cell population. The clonal B-cell population was identified only after back-gating based on cytoplasmic light chain analysis revealed a distinct subset of CD19-negative, CD38-negative cells with restricted λ light chain expression within the lymphocyte gate, prompting additional B-cell lymphoma panel testing. We emphasize that when a CD19-negative clonal B-cell population is encountered, LPL should remain in the differential diagnosis, particularly in the context of an IgG or IgA paraprotein and concordant light chain restriction between B cells and plasma cells.

According to the current International Consensus Classification (ICC), only IgM-type LPL is explicitly recognized as a distinct entity, with no specific provisions provided for non-IgM types (1). In contrast, the WHO-HAEM5 classification defines two LPL subtypes: the common IgM-type and the rare non-IgM type, which accounts for approximately 5% of cases. The latter includes cases with IgG or IgA paraprotein, non-secretory LPL, and IgM-type LPL without bone marrow involvement (2). Current literature regarding non-IgM LPL remains limited to small case series. A Swedish lymphoma registry study revealed that non-IgM LPL is frequently misdiagnosed, with a substantial proportion of cases reclassified as other lymphoma subtypes upon expert review, underscoring the diagnostic challenges of this rare entity (12). Japanese researchers reported no significant difference in *MYD88* L265P variant allele frequency between non-IgM LPL and WM (13). Conversely, a Korean retrospective study suggested that non-IgM LPL might exhibit more heterogeneous treatment responses and a higher 5-year mortality rate (14). Given its rarity, the clinicopathological characteristics and the biological relationship of non-IgM LPL to WM remain unclear.

The clinical spectrum of non-IgM LPL is increasingly recognized as heterogeneous. While most patients, including the present case, follow an indolent course, some may develop significant organ dysfunction. Regarding management, the Consensus Panel 4 from the 12th International Workshop on Waldenström’s Macroglobulinemia (IWWM-12) recommends classifying non-IgM LPL as a distinct subtype and suggests adhering to WM treatment strategies pending further prognostic data on *MYD88* status (15). To date, several cases of non−IgM LPL with *MYD88* L265P mutations have been treated with Bruton tyrosine kinase (BTK) inhibitors, demonstrating favorable responses in most instances (16, 17). Notably, none of those cases exhibited CD19 negativity. Our asymptomatic patient was appropriately managed with active surveillance. If treatment becomes necessary in the future, BTK inhibitors represent a promising therapeutic option for *MYD88*−mutated non−IgM LPL, including cases with atypical immunophenotypes.

In conclusion, we present a diagnostically challenging case of CD19-negative non−IgM LPL. The atypical immunophenotype, characterized by the loss of CD19 expression, complicated the initial diagnostic workflow. This case underscores that establishing a diagnosis for this rare entity necessitates a comprehensive synthesis of clinical presentation, morphological assessment, multiparameter flow cytometry, and molecular genetic profiling.

## Data Availability

The raw data supporting the conclusions of this article will be made available by the authors, without undue reservation.
